# Severe Hypertrophic Cardiomyopathy Caused by a Protein Kinase Adenosine Monophosphate-Activated Non-catalytic Subunit Gamma 2 (PRKAG2) Mutation With Refractory Chylous Effusions in a Neonate: A Case Report and Literature Review

**DOI:** 10.7759/cureus.72005

**Published:** 2024-10-21

**Authors:** Yohei Minamitani, Ayumi Oshima, Masayo Kanai, Yoichi Iwamoto, Hirotaka Ishido, Satoshi Masutani

**Affiliations:** 1 Department of Pediatrics, Saitama Medical Center, Saitama Medical University, Kawagoe, JPN

**Keywords:** chylous ascites, hypertrophic cardiomyopathy (hcm), lymphatic malformations, mtor inhibitors, prednisolone, prkag2, sirolimus

## Abstract

Protein kinase adenosine monophosphate-activated non-catalytic subunit gamma 2 (PRKAG2) cardiac syndrome is a rare genetic disorder characterized by hypertrophic cardiomyopathy and heart rhythm disturbances caused by mutations in the *PRKAG2* gene. Reports on PRKAG2 cardiac syndrome associated with refractory chylous effusion are extremely limited. Here, we present a neonatal case involving severe hypertrophic obstructive cardiomyopathy accompanied by chylous ascites and lymphatic malformations. The patient was diagnosed prenatally with hypertrophic cardiomyopathy. After birth, she developed severe respiratory failure, along with refractory chylous and pericardial effusions. Lymphoscintigraphy revealed lymphatic malformations in the right inguinal region. Prednisolone and sirolimus were administered to manage the chylous ascites and lymphatic malformations. Unfortunately, the patient succumbed to sepsis at two months of age. A de novo c.1592G>A (p.Arg531Gln) heterozygous variant of *PRKAG2* has also been identified. The association between *PRKAG2*, chylous effusion, and lymphatic malformations remains unclear. Further research is required to assess the effects and safety of prednisolone and sirolimus on chylous ascites in patients with PRKAG2 cardiac syndrome.

## Introduction

The protein kinase adenosine monophosphate-activated non-catalytic subunit gamma 2 (*PRKAG2*) gene encodes the γ2 subunit of adenosine monophosphate-activated protein kinase (AMPK), regulating cellular energy homeostasis [1]. Mutations in the *PRKAG2* gene cause chronic activation of AMPK, leading to glycogen accumulation in cells [2]. PRKAG2 cardiac syndrome is a rare autosomal dominant genetic disorder characterized by ventricular preexcitation, supraventricular arrhythmias, and cardiac hypertrophy due to glycogen accumulation in the myocardium [1,2]. In severe cases, patients show fetal or neonatal hypertrophic cardiomyopathy with a lethal prognosis [3]. However, there are very few reports of PRKAG2 cardiac syndrome associated with refractory chylous effusion. Here, we report a rare neonatal case of severe hypertrophic cardiomyopathy caused by PRKAG2 cardiac syndrome, presenting with refractory chylous ascites and lymphatic malformations.

## Case presentation

The patient's mother was a healthy 27-year-old Asian female, gravida 4 para 3, with no family history of cardiac disease. Fetal ultrasonography performed at 25 weeks of gestation revealed biventricular hypertrophy, pericardial effusion, and bradycardia. Pulmonary hypoplasia was suspected due to severe cardiomegaly and abdominal distension. Fetal hydrops and severe myocardial hypertrophy were worsening.

**Figure 1:**
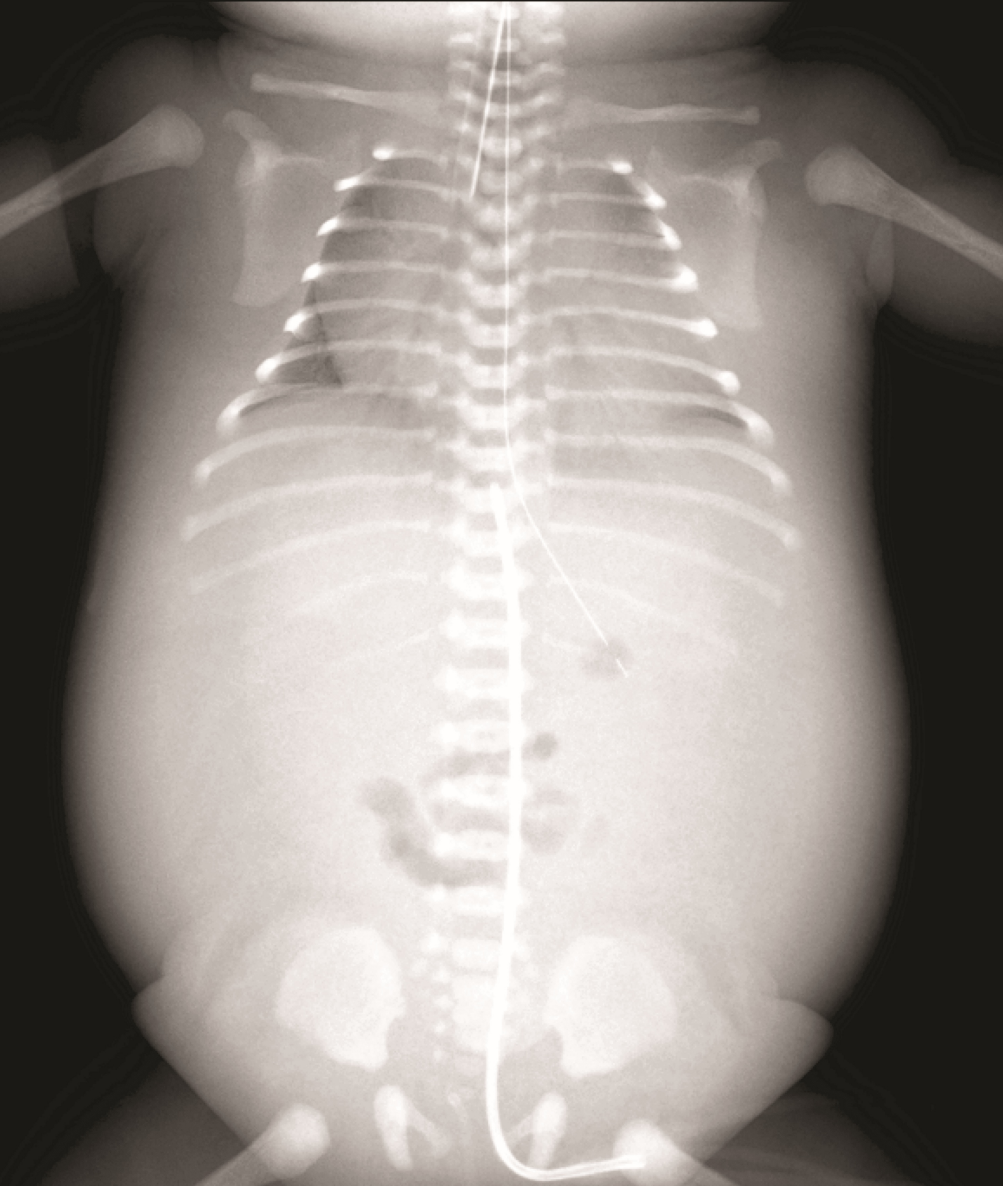
Chest and abdominal radiograph Chest and abdominal radiography performed on the first day of life revealed cardiomegaly, pulmonary hypoplasia, and marked systemic and pulmonary edema.

A female infant was delivered vaginally, at 34 weeks gestation, weighing 2,500 grams at birth. Soon after birth, surfactant therapy and mechanical ventilation were required, and inhaled nitric oxide therapy was initiated for persistent pulmonary hypertension of the newborn due to pulmonary hypoplasia (Figure 1).

**Figure 2:**
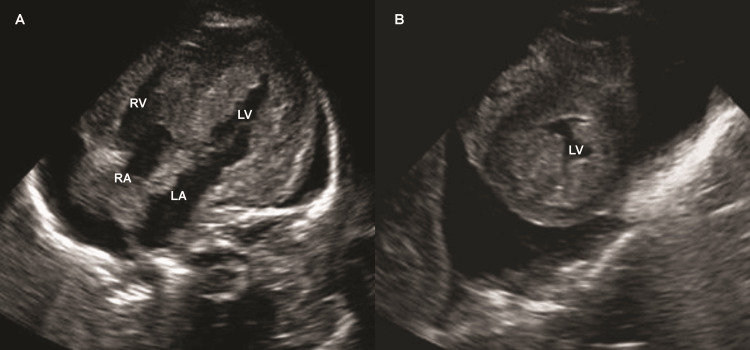
Transthoracic echocardiography Echocardiography in the apical four-chamber (A) and short-axis (B) views revealed severe ventricular hypertrophy and pericardial effusion. RV: right ventricle; RA: right atrium; LV: left ventricle; LA: left atrium

Echocardiography showed asymmetric myocardial hypertrophy, with a ventricular septal thickness of 13.0 mm (+22.2 SD) and a left ventricular posterior wall thickness of 7.7 mm (+10.6 SD). Left ventricular outflow tract blood flow was accelerated (2.2 m/s), leading to a diagnosis of hypertrophic obstructive cardiomyopathy (Figure 2). Electrocardiography revealed normal sinus rhythm, bradycardia, and a short PR interval.

**Figure 3:**
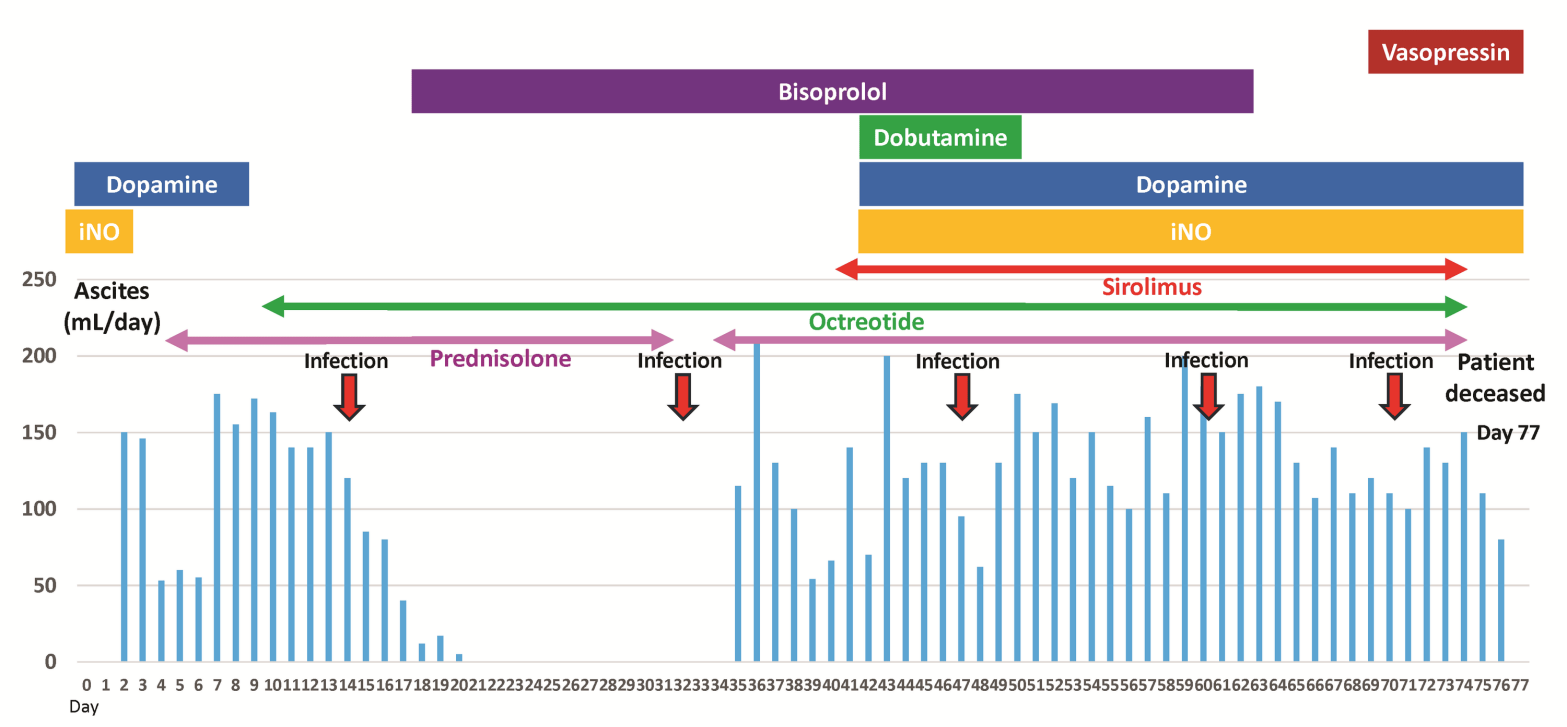
Clinical course The blue bar graph shows the daily volume of ascitic fluid in the abdominal drainage tube. Abdominal drainage was temporarily discontinued at 21 days of age. Ascites reaccumulated after the discontinuation of prednisolone at 31 days of age. The patient repeatedly developed bacterial infections (red arrow). Bisoprolol is also used to treat hypertrophic obstructive cardiomyopathy. At 42 days of age, dopamine, dobutamine, and inhaled nitric oxide were initiated for hypotension and hypoxemia due to worsening ascites and pericardial effusion. iNO: inhaled nitric oxide

Abdominal tube drainage was required from the first day of age, and ascitic fluid with lymphocytosis (lymphocytes, 92%) was diagnosed as chylous ascites. Despite total parenteral nutrition, the ascites persisted at approximately 50 mL/kg/day. After the initiation of prednisolone and octreotide, the ascites temporarily decreased, and abdominal tube drainage was paused at 21 days of age. However, ascites reaccumulated after the discontinuation of prednisolone at 31 days of age, necessitating re-drainage at 35 days of age (Figure 3).

G-band chromosome analysis of the patient revealed a normal karyotype. Although both hypertrophic cardiomyopathy and chylothorax are common in Noonan's syndrome [4], no variants have been identified in the genes related to Noonan syndrome (*PTPN11, SOS1, RAF1, RIT1, KRAS, NRAS, SHOC2, CBL, BRAF, SOS2, MRAS, RRAS, LZTR1, RRAS2, HRAS, MAP2K1, MAP2K2,* and *PPP1CB*) by a targeted next-generation sequencing panel testing. Pompe, Fabry, Gaucher, and Mucopolysaccharidosis may be the underlying diseases of hypertrophic cardiomyopathy [5], but the activity of each lysosomal enzyme is normal.

A large amount of chylous ascites of 50 to 200 mL/kg/day persisted, thus daily blood product transfusions were required for hypoalbuminemia and coagulopathy. Pericardial effusion gradually worsened. Lymphoscintigraphy performed at 40 days of age showed abnormal accumulation in the right inguinal region, leading to a diagnosis of lymphatic malformation. Sirolimus, a mechanistic target of rapamycin (mTOR) inhibitor, was initiated for refractory chylous ascites associated with lymphatic malformations at 41 days of age. After initiating sirolimus, the pericardial effusion significantly decreased. However, the chylous ascites and generalized edema did not improve. She developed severe hypoxemia and hypotension due to sepsis at 70 days of age and eventually died at 77 days of age. Next-generation whole exome sequencing of the patient and her parents revealed a pathogenic de novo c.1592G>A (p.Arg531Gln) heterozygous variant in the *PRKAG2* gene.

## Discussion

We present a rare case of severe hypertrophic cardiomyopathy with refractory chylous ascites that eventually identified a pathogenic mutation in the *PRKAG2* gene. To our knowledge, this is the first reported case of PRKAG2 cardiac syndrome with refractory chylous ascites and lymphatic malformations. Additionally, although prednisolone and sirolimus were partially effective in the management of hydrops in this case, these drugs may be related to the occurrence of sepsis, due to their immunosuppressive effects.

*PRKAG2* gene encodes the γ2-subunit of AMPK, regulating cellular energy homeostasis [6]. Certain mutations in *PRKAG2* cause the continuous activation of AMPK, excessive glucose uptake, and glycogen synthesis, leading to non-lysosomal glycogen storage in cardiac muscle cells [7]. The phenotypes of PRKAG2 cardiac syndrome vary, typically with ventricular pre-excitation and cardiac hypertrophy in adolescence and adulthood, and rarely with fetal- and infantile-onset severe hypertrophic cardiomyopathy [8].

In previous studies, p.Arg531Gln was reported to be a mutation in *PRKAG2* associated with lethal hypertrophic cardiomyopathy [9]. We systematically searched fetal and infantile cases in PubMed using following search strategy: ("Fetus"[Mesh] OR "Infant, Newborn"[Mesh] OR "Infant"[Mesh] OR fetus [TIAB] OR fetal [TIAB] OR neonat* [TIAB] OR newborn [TIAB] OR infant* [TIAB] OR baby [TIAB] OR babies [TIAB]) AND (PRKAG2 [TIAB] OR "AMP-Activated Protein Kinases"[Mesh] OR AMP-activated protein kinase [TIAB]) AND ("glycogen storage disease"[Mesh] OR "Cardiomyopathies"[Mesh] OR "Cardiomyopathy, Hypertrophic"[Mesh] OR cardiomyopathy [TIAB] OR hypertrophic cardiomyopathy [TIAB]). The initial database search identified 80 reports, and 70 reports were excluded after reviewing the titles and abstracts: 64 reports were basic science investigations, and six reports involved children and adults beyond the infantile period. Of these, 14 fetal or infantile cases from 10 reports were identified (Table 1) [3,6,7,9-15].

**Table 1:** Infantile-onset cardiomyopathy with PRKAG2 mutation *Same variant with our case HCM: hypertrophic cardiomyopathy; DCM: dilated cardiomyopathy; IVS: interventricular septum; PRKAG2: protein kinase adenosine monophosphate-activated non-catalytic subunit gamma 2

Author	Year	Sex	Gestational age	Presenting age	Clinical findings	Outcome	Mutation
Burwinkel et al. [10]	2005	Female	31 weeks	Fetus	Cardiomegaly, bradycardia, ventricular fibrillation	Died (2 months)	Arg531Gln (1592G>A)*
		Female	37 weeks	1st week of life	Fetal bradycardia, cardiac failure, severe hypertrophic cardiomyopathy	Died (34 days)	Arg531Gln (1592G>A)*
		Male	28 weeks	At birth	Bradycardia, biventricular hypertrophy, pericardial and pleural effusions, ascites	Died (21 days)	Arg531Gln (1592G>A)*
Akman et al. [3]	2007	Female	NA	10 weeks of life	Severe biventricular hypertrophy	Died (5 months)	Arg384Thr (1151G>C)
Kelly et al. [11]	2009	Male	NA	6 months of life	Systolic ejection murmur, left ventricular hypertrophy, HCM	Alive	Glu506Gln (NA)
Austin et al. [7]	2017	Male	38 weeks	2.5 months of life	Hypotonia, areflexia, feeding difficulties, mild hypertrophy of the IVS	Alive	Gly100Ser (298G>A)
Torok et al. [12]	2017	Female	Term	Fetus (27 weeks)	Mild hypertrophy of the IVS, HCM developmental delay	Alive	Lys475Glu (1423A>G)
		Female	Term	5 weeks of life	Severe HCM, ventricular ectopy, progressive cardiopulmonary failure	Died (4 months)	Arg531Gln (1592G>A)*
		Male	Term	2 months of life	Hypotonia, areflexia, mild hypertrophy of the IVS	Alive	Gly100Ser (298G>A)
Xu et al. [6]	2017	Female	NA	Fetus (27 weeks)	Modest HCM	Alive	Lys475Glu (1423A>G)
Gorla et al. [9]	2018	Male	36 weeks	Fetus (28 weeks)	Fetal hydrops, severe HCM small pericardial effusion	Died (7 weeks)	Arg531Gln (1592G>A)*
Beyzaei et al. [13]	2021	Female	NA	1 month of life	Pulmonary hypertension, short stature mitral, and tricuspid regurgitation	Alive	Met198Leu (592A>T)
Gong et al. [14]	2022	Female	NA	9 months of life	DCM with heart failure	Alive	Thr142Ile (425C>T)
White-Brown et al. [15]	2024	Male	35 weeks	Fetus (32 weeks)	Biventricular hypertrophy, bradycardia, large pleural effusion	Died (17 hours)	Arg384Gly (1150A>G)
Present case	2024	Female	34 weeks	Fetus (25 weeks)	Fetal hydrops, severe HCM refractory ascites, pericardial effusion	Died (77 days)	Arg531Gln (1592G>A)*

Of these, five cases showed a heterozygous c.1592G>A (p.Arg531Gln) mutation in *PRKAG2*, and all of them died within several months of age. The mutation of p.Arg531Gln is associated with the lethal phenotype of PRKAG2 cardiac syndrome. The poor clinical prognosis of the present case with p.Arg531Gln is consistent with that of previous reports.

However, the association between the p.Arg531Gln mutation, refractory chylous ascites, and lymphatic malformations remains unclear. To the best of our knowledge, this is the first reported case of PRKAG2 cardiac syndrome with refractory chylous ascites and lymphatic malformations. Further studies are required to assess the relationship between *PRKAG2*, chylous ascites, and lymphatic malformations.

Generally, the management of neonatal chylous effusion includes medium-chain triglyceride (MCT) formulas, total parenteral nutrition, and octreotide [16]. Prednisolone is a useful pharmacological treatment for chylous effusions [17,18]. In the present case, chylous ascites significantly worsened after the discontinuation of prednisolone; thus, we assessed whether prednisolone was effective in our patient. Recent studies have reported the effectiveness of sirolimus in treating chylous effusions caused by lymphatic malformations [19]. In the present case, although the pericardial effusion improved after the initiation of sirolimus, the chylous ascites did not improve. Moreover, due to their immunosuppressive effects, prednisolone and sirolimus may be associated with the occurrence of sepsis.

## Conclusions

PRKAG2 cardiac syndrome caused by the p.Arg531Gln mutation may present a lethal prognosis. Further studies are required to investigate the pathophysiological association between chylous effusions and lymphatic malformations and to determine the effects and safety of immunosuppressive therapies for chylous effusions in patients with PRKAG2 cardiac syndrome.
